# Automated Video Quality Assessment for the Edinburgh Visual Gait Score (EVGS)

**DOI:** 10.3390/mps8040071

**Published:** 2025-07-03

**Authors:** Rajkumar Arumugam Jeeva, Edward D. Lemaire, Ramiro Olleac, Kevin Cheung, Albert Tu, Natalie Baddour

**Affiliations:** 1 Department of Mechanical Engineering, University of Ottawa, Ottawa, ON K1N 6N5, Canada; 2 Faculty of Medicine, University of Ottawa, Ottawa, ON K1H 8M2, Canada; elemaire@uottawa.ca; 3 Department of Neuroorthopeadics, Division of Surgery, Nicolas Avellaneda Hospital, San Miguel de Tucuman 4000, Tucuman, Argentina; ortopediainfantiltucuman@gmail.com; 4 Division of Plastic Surgery, Department of Surgery, Children’s Hospital of Eastern Ontario, Ottawa, ON K1H 8L1, Canada; kcheung@cheo.on.ca; 5 Division of Neurosurgery, Department of Surgery, Children’s Hospital of Eastern Ontario, Ottawa, ON K1H 8L1, Canada; atu@cheo.on.ca; 6 Department of Surgery, University of Ottawa, Ottawa, ON K1N 6N5, Canada

**Keywords:** gait analysis, video quality assessment, pose estimation, random forest, edinburgh visual gait score

## Abstract

This research addresses critical challenges in clinical gait analysis by developing an automated video quality assessment framework to support Edinburgh Visual Gait Score (EVGS) scoring. The proposed methodology uses the MoveNet Lightning pose estimation model to extract body keypoints from video frames, enabling detection of multiple persons, tracking the person of interest, assessment of plane orientation, identification of overlapping individuals, detection of zoom artifacts, and evaluation of video resolution. These components are integrated into a unified quality classification system using a random forest classifier. The framework achieved high performance across key metrics, with 96% accuracy in detecting multiple persons, 95% in assessing overlaps, and 92% in identifying zoom events, culminating in an overall video quality categorization accuracy of 95%. This performance not only facilitates the automated selection of videos suitable for analysis but also provides specific video improvement suggestions when quality standards are not met. Consequently, the proposed system has the potential to streamline gait analysis workflows, reduce reliance on manual quality checks in clinical practice, and enable automated EVGS scoring by ensuring appropriate video quality as input to the gait scoring system.

## 1. Introduction

Gait analysis is an important tool in clinical settings for diagnosing and treating various movement-related pathologies [1]. The systematic study of human locomotion provides invaluable insights into biomechanical stability and motor function, supporting surgical planning and rehabilitation therapies [2]. While traditional gait analysis methods have relied on manual observation and scoring, recent advancements have led to automated systems for recognizing gait events and estimating gait parameters [3,4]. However, the effectiveness of these automated systems heavily depends on the quality of input videos. Video quality assessment (VQA) has become increasingly important in the context of medical imaging and gait analysis [5]. Unlike entertainment or general-purpose videos, medical videos require specialized quality metrics that consider diagnostic relevance and clinical utility. In the realm of gait analysis, video quality affects the accuracy of automated assessments, making it crucial to develop robust VQA methods tailored to this domain [5,6].

Content-based video quality assessment has evolved to incorporate both technical and semantic elements, recognizing that quality extends beyond mere technical parameters [7,8]. Video quality assessment often focus on the technical viewpoint, assessing video distortions (i.e., blurring, artifacts) and their effects on quality to compare and direct technological systems like cameras [9], restoration algorithms [10], and compression standards [11]. Clear-textured videos should be noticeably higher quality than grainy video. However, a number of recent studies found that human quality evaluation of movies was also influenced by preferences for non-technical semantic elements such as composition and content [12]. The content recommendation systems on websites like YouTube or TikTok consider videos with meaningful or highly relevant content as high quality even if the visual clarity of the video is blurry or low resolution [13]. In entertainment videos, viewers are more tolerant of poor technical quality (e.g., low frame rate) if they are highly interested in the content [14]. Medical images/videos are typically large and can be compressed to efficiently manage storage and databases. When compressing medical images/videos, it is important to ensure that all the features, like anatomical structures and textures, are retained. Diagnostic image quality is not solely determined by the image’s visual clarity but also by the essential features required for diagnosis [6]. The underlying paradigm is to quantify the quality of medical content by its effectiveness with respect to its intended purpose [5,15]. A study [16] included a segmentation-based quality assessment framework for retinal images that used unsupervised vessel segmentation and then extracted features from the binary segmentation image for classifying the quality of an image using Support Vector Machine (SVM) and ensemble decision tree classifiers. Pixel-wise binary segmentation based on AdaBoost with local texture descriptors was used to compare the Dice overlap coefficient with manual segmentations to a variety of quality metrics [17]. A CNN was used to segment the macular region in eye fundus images for image quality assessment, where good image quality was when the macular area was above a threshold [18].

The presence of artifacts like zoom effects, more than one person, or an invalid camera angle in input video can severely affect automated gait analysis algorithm performance. Recent research has focused on developing task-specific quality assessment methods for medical videos [7]. These approaches aim to evaluate video quality based on its fitness for specific clinical tasks, such as gait parameter estimation or joint angle measurement. By incorporating domain knowledge and clinical requirements, these methods provide more relevant quality scores than traditional, general-purpose VQA techniques [5,6,9].

The Edinburgh Visual Gait Score (EVGS) [19] is a clinical tool for scoring pathological gait using videos in the coronal and sagittal planes of the person walking. EVGS is typically visually assessed by experts with experience in gait analysis and could include tools to step through the video frame-by-frame. Automated EVGS calculates 17 EVGS parameters and an overall score by using AI-based pose detection, novel algorithms to calculate input variables (stride events, joint angles, etc.), and output scores [3,4]. Automated EVGS depends not only on good overall video quality but also on proper video capture. Therefore, an automated video quality assessment framework capable of detecting zoom artifacts, multiple people, and correct plane orientation is essential to ensure appropriate input for automated EVGS scoring, eliminating the need for manual video quality checks.

This research encompassed the development and validation of a comprehensive video quality assessment framework specifically designed for gait analysis videos. The framework addresses key challenges in automated gait analysis, including detecting multiple persons, plane orientation classification, zoom artifact identification, and resolution assessment. By providing an objective measure of video quality and generating feedback to users, this approach seeks to eliminate the need for manual quality checking, making automated gait analysis scoring truly automatic. Currently, a validated method for automatic video quality scoring is not available for gait-related videos.

## 2. Development Criteria

To ensure that appropriate videos are available for automated EVGS scoring, an automated video quality scoring system is needed. Ideally, this video quality assessment should occur immediately after the video is taken, so that feedback can be provided to the person taking the video in cases where the quality is poor.

Based on experience with previous development of an automated EVGS system [3,4], various video quality requirements were identified. These included:

- Multiple people detection,
- Multiple people overlap detection,
- Plane detection,
- Zoom artifact detection,
- Extract video segments without zoom,
- Resolution detection,
- Overall quality classification and feedback generation.

The video quality assessment objective in this research is related to gait videos for analysis using the EVGS tool. Video quality requirements include an appropriate plane of movement, appropriate resolution, identification of multiple and overlapping people, and camera zooming. Novel algorithms were developed to address these requirements.

## 3. Development and Validation Datasets

The algorithm was developed and validated using a dataset of videos from the Sanatorio del Norte medical center in Tucumán, Argentina (private deidentified dataset, approved for secondary use. This study was conducted according to the guidelines of the Declaration of Helsinki and approved by the Research Ethics Board of the Children’s Hospital of Eastern Ontario, protocol code: CHEOREB# 22/133X). This dataset included gait videos in sagittal, coronal, and transverse planes of 230 individuals with walking disorders. Recording was set up in a straight 30-foot (10 m)-length walkway. Videos were recorded at 60 Hz and captured in a closed environment featuring bright and diffuse lighting to ensure that key anatomical landmarks remained visible throughout the gait cycle. Recordings were made against a plain, light-colored background to enhance participant visibility.

Coronal plane videos were captured using a tripod-mounted mobile device, positioned to face the person walking along the walkway. The stationary setup ensured a stable view and eliminated camera shake. The tripod height was adjusted so that the person remained within the center of the camera frame, occupying at least two-thirds of the field of view throughout the walk. In some cases, a camera operator manually controlled the device during recording to maintain optimal framing and focus as the person approached the camera.

Sagittal plane videos were recorded using a gimbal-mounted mobile device handled by a trained operator who walked parallel to the person. This approach allowed the camera to track the participant in motion, keeping them centered within the frame across all strides and minimizing parallax distortion. The device was held at knee level to ensure accurate perspective on all major body segments, including lower and upper limbs and the trunk. The sagittal recordings were captured in portrait orientation to allow the camera to remain closer to the person while maintaining full-body visibility.

Transverse plane videos were acquired from an overhead perspective using a mobile device mounted on a gimbal and the operator on a stepladder. This configuration allowed for visualization of foot placement and gait progression patterns that are difficult to capture from coronal or sagittal views. To assist in visualizing foot contacts and tracking stride width and orientation, the floor was covered with a black vinyl rug and dusted with talcum powder, which highlighted the footfalls of each person. This vertical video capture was conducted simultaneously with sagittal and coronal recordings, requiring coordination among multiple trained camera operators.

The dataset was divided into development and validation datasets, with 161 videos in the development set and 69 videos in the validation dataset. The first 161 videos in the overall dataset were allocated to development. The number of videos for validation differed for each video quality requirement, since each item addressed different video criteria. Therefore, each subsection that describes the framework component also defines the dataset used to validate the specific component.

## 4. Pose Detection

Movenet Multipose Lightning [20] was chosen as the pose estimation model to extract keypoints. The architecture consists of a MobileNetV2 image feature extractor with Feature Pyramid Network decoder followed by CenterNet prediction heads with custom post-processing logics. The pose estimation model output is a float32 tensor (1, 6, 56) with the following properties.

**Figure 1:**
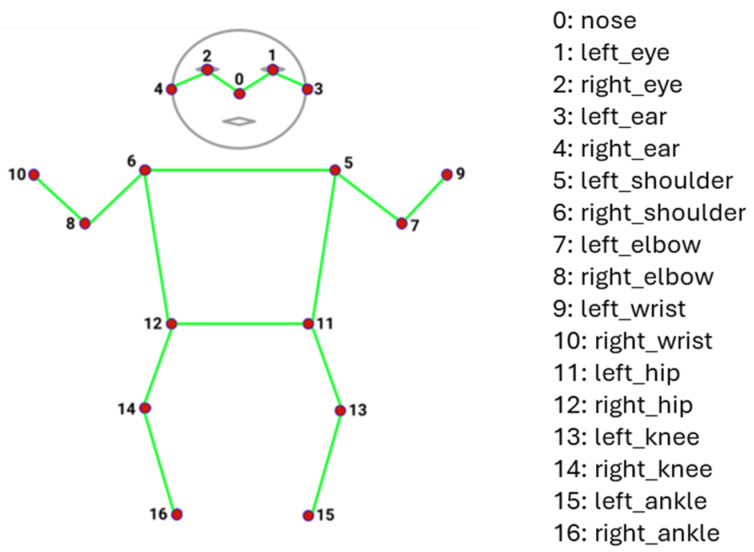
Movenet lightning multipose keypoints.

- The first dimension is the batch dimension, which is always equal to 1.
- The second dimension corresponds to the maximum number of instance detections. The model can detect up to six people in the image frame simultaneously.
- The third dimension represents the predicted bounding box/keypoint locations and confidence scores.
- The 17 keypoints include nose, left eye, right eye, left ear, right ear, left shoulder, right shoulder, left elbow, right elbow, left wrist, right wrist, left hip, right hip, left knee, right knee, left ankle, and right ankle [20]. These keypoints are shown in Figure 1.

## 5. Video Quality Assessment Framework

**Figure 2:**
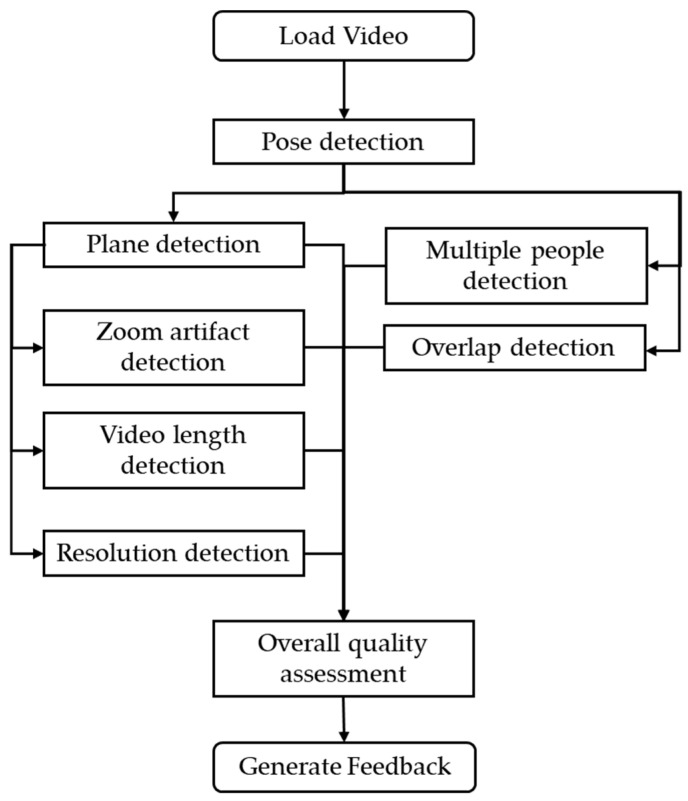
Video quality framework.

The video quality assessment framework begins by loading the gait video and applying the MoveNet Multipose Lightning pose estimation model to detect all individuals and extract their skeletal keypoints frame-by-frame. These keypoints are used to compute various quality-related metrics. The framework performs quality analyses for each subcategory and then combines these subcategory scores into an overall score (Figure 2).

For multiple people detection, if more than one person is identified, the user is prompted to select the target patient, and the algorithm tracks this person across all subsequent frames using centroid-based Euclidean distance matching. Multiple people overlap detection involves checking for overlap between bounding boxes, flagging frames where other individuals obstruct the target subject.

Plane detection classifies the video as sagittal, coronal, or transverse based on geometric ratios calculated from shoulder and hip keypoints. This ensures that only videos captured from clinically appropriate views are considered valid. Plane detection is followed by zoom artifact detection, in which the algorithm checks for variations in the bounding box height of the target person across frames. Sudden changes are flagged as zoom events, and by video length detection, the algorithm segments the video part without zoom and also checks that the segmented video is greater than the minimum duration required for the automated EVGS algorithm. Additionally, resolution detection is carried out by converting normalized bounding box coordinates into pixel values and verifying that the person’s size exceeds a defined threshold.

Overall video quality is classified from all computed metrics and a random forest classifier. Video quality labels are “Good videos” that are suitable for automated EVGS algorithm and “Manual edit required” that require manual edit before EVGS processing. When a video fails the quality check, the algorithm also generates automated feedback based on the individual metric score to guide the user, aiming to improve the video acquisition process and reduce the need for manual quality checks.

### 5.1. Multiple People Detection

**Figure 3:**
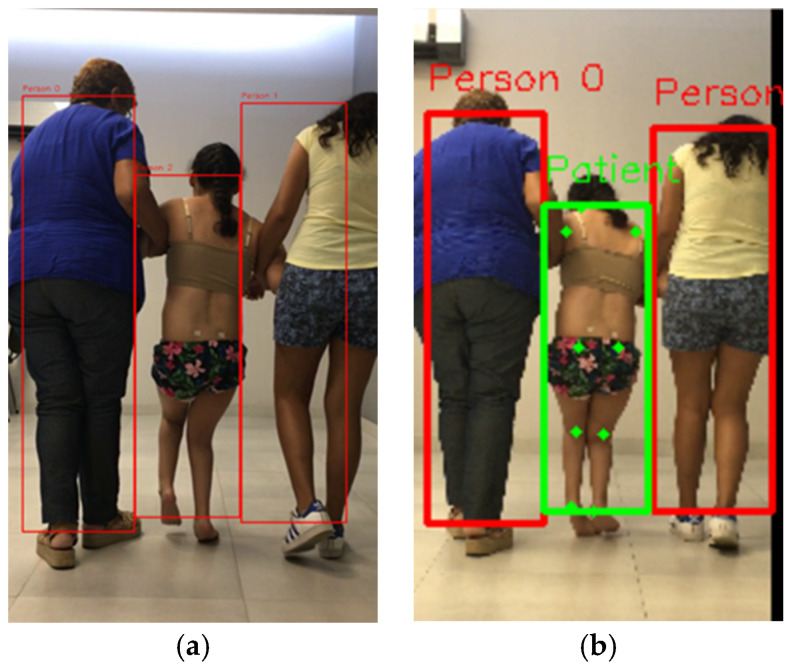
User interface to (**a**) select patient and (**b**) track the selected patient.

Output from MoveNet Multipose Lightning provides bounding boxes for each person in the video. Therefore, we know the number of people in the video by counting the number of bounding boxes. A bounding box is a rectangular area that covers the person, defined with *x*_1_, *y*_1_, *x*_2_, and *y*_2_ coordinates. The pose estimation model also assigns a confidence score to each detected person in the frame, which indicates the detection quality. Detections with a confidence score less than a defined minimum value (default value is 0.5) are considered lower reliability detections. Lower reliability detections are eliminated from further analyses. If the algorithm finds more than one person, the user is prompted to select the person of interest in that frame. This selection is made possible through a user interface where the user can see the frame with overlaid red bounding boxes and keypoints for each detected person (Figure 3). If the frame does not show the target person, the user has the option to skip to the next frame with more than one detected person.

Once selected, the algorithm checks the chosen person’s keypoints in future frames to ensure that the same person is selected from the current frame to the end of the video. A similarity measure based on the Euclidean distance between the centroid of the target person’s bounding box in the previous frame and the centroid of all bounding boxes in the current frame is used to determine the closest match.

(1) $$d=\min\left(\sqrt{{\left(x_{t}-x_{i}\right)}^{2}+({y_{t}-y_{i})}^{2}\;\;}\right),\;\forall i\;\in \;\{1,2,\ldots ,n\}$$

In Equation (1), (*x_t_*,*y_t_*) is the target person’s centroid in the previous frame, (*x_i_*,*y_i_*) is the centroid of the *i*th bounding box of current frame, and *n* represents number of people detected by the pose estimation model. The variable *d* represents the centroid Euclidean distance between the target patient’s bounding box in the previous and current frame. The bounding box in the current frame whose centroid has the smallest Euclidean distance from the centroid of the target patient’s bounding box in the previous frame is considered to be the target patient’s bounding box in the current frame. As a visual aid, the selected person has a green bounding box to differentiate this person from other detected people with red bounding boxes in the frame (Figure 3).

The multiple persons quality metric is the percentage of the number of frames with only the target patient’s bounding box relative to the total number of frames. This metric provides an overall view of how many frames have multiple people interfering in the video. A higher score indicates fewer multiple persons, thereby improving the suitability of the video for automated EVGS scoring.

### 5.2. Multiple People Overlap Detection

Overlap frames are where the target person is occluded by another person in the video. Overlap can be identified by bounding box coordinates (*x*_1_, *y*_1_, *x*_2_, *y*_2_) of detected person in the frame. Here, *x*_1_ is the left edge, *y*_1_ is the top edge, *x*_2_ is the right edge, and *y*_2_ is the bottom edge of the bounding box. The conditions to check overlap are:

- *x*_1_ of the target person bounding box is less than *x*_2_ of any other bounding box in the frame, and *x*_2_ of the target person bounding box is greater than *x*_1_ of any other bounding box in the frame.
- *y*_1_ of the target person bounding box is less than *y*_2_ of any other bounding box in the frame, and *y*_2_ of the target person bounding box is greater than *y*_1_ of any other bounding box in the frame.

If one of the above conditions is true, then the frame has overlapping bounding boxes, and this frame is flagged as an overlapping frame. Overlap detection is repeated for all the frames in the video. The quality metric for multiple people overlap is the percentage of the number of overlapping frames to the total number of frames.

### 5.3. Plane Detection

Plane identification is a very important aspect of gait video quality assessment, since EVGS outcome measures are related to either the sagittal or coronal plane [3]. The plane detection algorithm classifies each video as the sagittal, coronal, or transverse plane based on the body’s geometric orientation. The first step in plane detection is to extract left shoulder, right shoulder, left hip, and right hip keypoints in every video frame. To assess the video perspective and determine the plane of observation, three geometric ratios are derived based on the selected keypoints.

Horizontal Shoulder Distance (HSD): A measure of upper body width in the frame, measured across the horizontal distance between the left and right shoulders. This gives insight into the body orientation in the horizontal plane and is computed using Equation (2).

*D_s_* =|*x_ls_* − *x_rs_*| (2)

where *D_s_* is the horizontal distance between right and left shoulders, *x_ls_* is the x coordinate of the left shoulder, and *x_rs_* is the x coordinate of the right shoulder.

Right Vertical Shoulder-to-Hip Distance (RVSD): Vertical positioning of the right side of the body in relation to the frame, as the vertical area between the right shoulder and the right hip (Equation (3)).

*D_rsh_* = |*y_rs_* − *y_rh_*| (3)

where *D_rsh_* is the vertical distance between the right shoulder to right hip, *y_rs_* is the x coordinate of left shoulder, and *y_rh_* is the x coordinate of the right hip.

Left Vertical Shoulder-to-Hip Distance (LVSD): Vertical positioning of the left side of the body within the frame, as the distance between the left shoulder and left hip vertically (Equation (4)).

*D_lsh_* = |*y_ls_* − *y_lh_*| (4)

where *D_lsh_* is the vertical distance between the left shoulder to left hip, *y_ls_* is the y coordinate of left shoulder, and *y_lh_* is the y coordinate of the left hip.

Additional metrics are also computed to understand the body orientation and to differentiate between sagittal and coronal views. The Initial Ratio (*r*_1_) is the ratio between HSD and RVSD (Equation (5)). The Second Ratio (*r*_2_) is the ratio between LVSD and HSD (Equation (6)), and the Final Ratio (*R*) is the ratio of *r*_2_ and *r*_1_ (Equation (7)).

*r*_1_ = *D_s_*/*D_rsh_* (5)

*r*_2_ = *D_lsh_*/*D_s_* (6)

*R* = *r*_2_/*r*_1_ (7)

Videos where the person is walking in one direction can be classified using *R* (i.e., right to left or left to right in sagittal videos, towards camera or away from camera in coronal videos). However, if the person is walking in the sagittal plane, *R* changes when they turn around, potentially leading to misclassification. In addition, the ratios computed over all frames may have extreme values affected by either noise or poorly detected keypoints. Therefore, to make the method robust, outlier filtering was implemented by applying the interquartile range.

- Calculate 25th (P25) and 75th (P75) percentiles of the ratio list (*R*).
- Ratios outside the P25 to P75 range are excluded.

The ratio list without outliers is termed the “Filtered Ratio List”, and the mean of the Filtered Ratio List is used to classify the video’s general body orientation.

**Figure 4:**
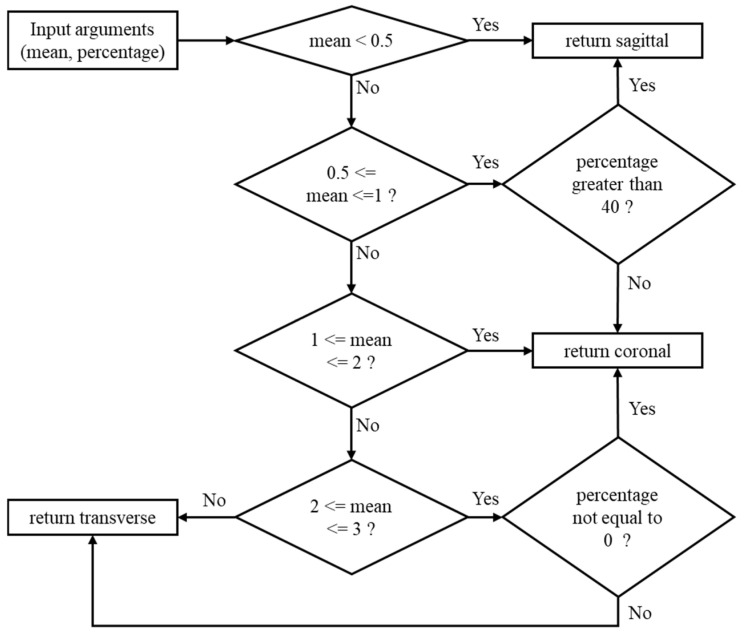
Classification criteria for plane classification. Mean refers to the average of the Filtered Ratio List. Percentage indicates the percentage of low ratios in the Filtered Ratio List.

Values in the Filtered Ratio List that are less than 0.7 are termed “low ratios”. The 0.7 threshold was determined empirically through a grid search optimization process, ensuring this threshold effectively distinguishes sagittal, coronal, and transverse views across diverse video datasets. The percentage of low ratios in the Filtered Ratio List was calculated by summing the number of frames with R below 0.7 and dividing this by the total number of frames in the Filtered Ratio List. Figure 4 shows the classification flow chart for plane identification.

If the mean of the Filtered Ratio List is below 0.5, or if the mean of the Filtered Ratio List is between 0.5 to 1 and the percentage of low ratios is more than 40, the body was oriented sideways relative to the camera position (sagittal plane).

If the mean of the Filtered Ratio List is between 0.5 and 1 and the percentage of low ratios is less than 40, or the mean of the Filtered Ratio List is between 1 to 2, or the mean of Filtered Ratio List is between 2 to 3 and the percentage of low ratios is not equal to 0, body orientation is in front or back facing the camera (coronal plane).

If the mean of the Filtered Ratio List is 2 or more, or the mean of Filtered Ratio List is between 2 to 3 and the percentage of low ratios is equal to 0, the plane is transverse (top view).

### 5.4. Zoom Artifact Detection

Zoom artifact detection is important since abrupt changes in the scale may introduce pose estimation and parameter errors. This section identifies sudden changes in bounding box height around the person of interest. The bounding box coordinates (*x*_1_, *y*_1_, *x*_2_, *y*_2_) are used to calculate bounding box height (Equation (8)).

*h* = *y*_2_ − *y*_1_ (8)

where *h* is the height of the bounding box, and *y*_1_ and *y*_2_ are y coordinates of the top left and bottom right of the bounding box. Since video resolutions can be very different, height is normalized by video frame height, and normalized height of the bounding box of all the frames in the video is stored in the variable “normalized_height”. This ensures the algorithm will be resolution-independent, thus capable of handling videos of any aspect ratio and resolution. Frame height data is usually variable because of minor inaccuracies in pose detection or slight postural changes by the person. A Gaussian filter with a kernel standard deviation of 2 is applied to the normalized height data to provide a clean height profile suitable for sharp variation detection and to prevent false positives. Once the algorithm smoothed height data, height changes between frames are calculated based on fixed step sizes (s). The smoothed height difference for every *i*th frame is given in Equation (9).

∆*h_i_* = *S*_*i*+*s*_ − *S_i_* (9)

where Δ*h* is the change in height, and *S* is the smoothed height data. The step-size difference approach ensures that only relevant changes in height are considered and, in this way, highlights major zoom events rather than gradual fluctuations. In the implementation, where the video frame rate is 60 Hz, *s* = 8 provides a good trade-off between sensitivity and robustness. The height differences are then compared to a threshold value *τ* = 0.05 to determine if a frame represents a zoom event. A Zoom-In event is identified when Δ*h* > *τ*, since the height increases suddenly. Frames with Δ*h* < −*τ* are defined as Zoom-Out, due to the fast decrease in height.

### 5.5. Extract Video Segments Without Zoom

This section explains how the algorithm systematically segments and scores usable video segments for gait analysis, considering both coronal and sagittal planes. For use with EVGS, the system ensures that no zoom event has occurred and that the video segment contains at least two walking strides. Each plane is analyzed independently to account for the unique characteristics of the respective planes.

#### 5.5.1. For Coronal Videos

The algorithm starts with segmenting the input video based on the change in the person’s height. Segmentation is cutting the video based on detecting sign changes in the height difference, as given in Equation (9). A new segment is created when the sign of Δ*h* changes. That is, one segment would be generated with every transition from an increasing to a decreasing height or decreasing to increasing height. For the videos without zoom, a segment is generated only when the person’s position relative to the camera changes. Specifically:

- If the person is walking away from the camera, a new segment is created when they turn and start walking toward the camera.
- If the person is walking toward the camera, a new segment is created when they turn and start walking away from the camera.
- If there is a zoom event, then a segment will also be generated when zooming in (patient height will increase) and zooming out (patient height will decrease).

For each segment, the algorithm detects the person’s orientation to the camera. Orientation here refers to the direction the person is facing, whether they are facing toward the camera (front) or away from the camera (back). The orientation is decided by shoulder alignment analysis, using the x-coordinates of the right and left shoulder keypoints. When the left shoulder keypoint is to the right of the right shoulder keypoint (i.e., left shoulder > right shoulder x-coordinate), the person is oriented with their front towards the camera. For orientation back to the camera, the left shoulder keypoint would be to the left of the right shoulder keypoint (i.e., left shoulder < right shoulder x-coordinate). In general, when the person is walking towards the camera, their height will gradually increase, and when the person walks away from camera, their height will decrease.

The algorithm eliminates video segments with a zoom event, where video segments with zoom are identified by the following conditions:

- Person walking towards the camera and height of the person is decreasing.
- Person walking away from the camera and height of the person is increasing.

Video segments with a duration less than three seconds (i.e., approximation for the time used to complete two strides) were rejected since two strides is the minimum requirement for automated EVGS analysis.

After eliminating video segments with less than three seconds and video segments with zoom, the remaining video segments are considered valid. If the number of frames between two valid video segments is less than five frames (i.e., difference of end frame number of a valid video segment and start frame number of the next video segment is less than five), the two video segments are merged into one valid video segment. The merged valid video segment has a start frame number from the first video segment and end frame number from the next valid video segment. This merging method maintains gait cycle continuity. These methods ensure that only video segments suitable for automated gait analysis are preserved.

All valid video segments are scored for quality, with scores ranging from 0 to 100. The duration of each valid video was extracted. Moderate intensity walking is 100 steps/minute; therefore, the approximate time to complete five steps is 8 s (five steps ensures that two viable strides are available for analysis). Thus, video segments with a minimum 8 s duration are awarded a maximum quality score (100), and video segments of 3 s or less are scored as 0. For video durations between 3 and 8 s, the score is scaled proportionally from 0 to 100 based on the segment’s length relative to the 0–8 s scale. Two of the highest scores are selected: one related to the segments where the person is oriented towards the camera, and another where the person is oriented away from the camera. Finally, general video quality is scored by computing the percentage of frames that belong to valid segments with respect to the total number of frames in the video.

#### 5.5.2. For Sagittal Videos

To classify video segments in the sagittal orientation (i.e., person walking from left to right or right to left), x-coordinates of the ears and nose keypoints are used. Since the coordinate system for the image starts from the top left, with increasing values to the right for the x-coordinate:

- If the x-coordinate of the ears is greater than the nose x-coordinate, the person is walking from right to left (i.e., ears are always to the right of the nose).
- If the x-coordinate of the ears is less than the nose x-coordinate, the person is walking left to right (i.e., ears are left of the nose).

One video segment is generated for every transition from left to right to right to left, or from right to left to left to right. In each video segment, the person is either walking left to right or right to left, not both.

Video segments are analyzed to detect zoom events. Unlike the coronal plane, the person’s height in the sagittal plane remains relatively constant, since the individual stays a similar distance from the camera. A zoom event in a video segment is identified using the approach in Section 5.4. As in Equation (9), Δ*h* represents the difference in bounding box height between frame *i* and frame *i* + 8. If Δ*h* exceeds 0.05, the frames from *i* to *i* + 8 are identified as zoom event frames and are eliminated. This elimination process is repeated at all instances where Δ*h* > 0.05, removing all zoom events and resulting in video segments free of zoom events. After this process, video segments with a duration less than three seconds, an approximation for the time used to complete two strides, were rejected since two strides is the minimum requirement for automated EVGS analysis. After removing video segments with duration less than 3 s, the remaining video segments are considered valid.

Sagittal plane video scoring follows the same method as coronal plane videos, where each video segment is scored based on duration. Scoring to assess zoom in sagittal video includes the highest score of left-to-right video segments, highest score of right-to-left video segments, and percentage of valid frames in the video.

### 5.6. Resolution Detection

The resolution score begins by extracting the bounding box coordinates of the person of interest from the pose estimation model. The pose estimation model provides normalized coordinates for the bounding box in the range of [0, 1]. These normalized coordinates are converted to pixel coordinates using the video frame resolution. The normalized bounding box coordinates (*x*_1_, *y*_1_, *x*_2_, *y*_2_) are converted using Equations (10) and (11):

*x_p_*_1_ = *x*_1_ *f_w_*, *x_p_*_2_ = *x*_2_ *f_w_* (10)

*y_p_*_1_ = *y*_1_
*f_h_*, *y_p_*_2_ = *y*_2_
*f_h_* (11)

where *x_p_*_1_, *y_p_*_1_, *x_p_*_2_, and *y_p_*_2_ are the pixel coordinates of the bounding box, and *f_h_* and *f_w_* are frame height and frame width, respectively. Then, the width and height of a person’s bounding box can be calculated using Equations (12) and (13).

*b_w_* = *x_p_*_2_ − *x_p_*_1_ (12)

*b_h_* = *y_p_*_2_ − *y_p_*_1_ (13)

where *b_w_* is the width of bounding box, and *b_h_* is the height of the bounding box. The calculated width and height are stored for each frame. The automated EVGS algorithm [3] uses the Openpose pose estimation model to extract keypoints with which the study calculated EVGS scores. From [21], if the width is less than 60 pixels and height is less than 80 pixels, then Openpose struggles to estimate keypoints, and the percent of missing keypoints increased more than 84%. Hence, frames with a bounding box resolution less than these thresholds are flagged as low-resolution frames. The final resolution score is the percentage of frames with an acceptable resolution using Equation (14).

*r_s_* = (1− *t_lrf_*/*t_f_*) ∗ 100 (14)

where *r_s_* is the resolution score, *t_lrf_* is the total number of low-resolution frames, and *t_f_* is total number of frames. This score provides a quantitative measure of the person of interest’s resolution quality in the video, helping to assess whether the input video meets the resolution requirements for automated gait analysis.

### 5.7. Overall Quality Classification

The objective of the quality classification is to classify the gait video as “good”, “manual edit required”, or “in the transverse plane”. Good videos can directly be used by automated EVGS algorithms. Manual edit required videos need to be edited (trimming, cropping, etc.) before they can be used in the automated EVGS algorithm. Since automated EVGS processes only sagittal and coronal planes, videos taken from the transverse plane were identified using the plane detection methodology and excluded from automated EVGS processing.

A random forest classifier is used to classify the coronal and sagittal videos as “good” or “manual edit required” videos. Gait videos from the development dataset were used to train the random forest classifier, and videos from the validation dataset were used to validate random forest classifier performance. Quality scores were calculated for all the videos in the development dataset. The classification framework used the calculated scores as input parameters for the random forest classifier: multiple persons (percent of frames without multiple people), multiple persons overlap (percent of frames without overlapped bounding boxes), zoom detection (1 if zoom occurred, 0 if no zoom), extracted clip without zoom (maximum score for video segment with 8 s or more), and resolution (percent of frames with acceptable resolution).

Hyperparameters such as number of estimators, maximum depth, minimum samples required to split a node, minimum number of samples required at leaf node, and bootstrap sampling of random forest classifier were tuned using the grid search approach from scikit-learn. The grid search approach calculates performance metrics such as accuracy, precision, sensitivity, specificity, and F1 score for all combinations of hyperparameters and returns the best hyperparameters. The selected hyperparameters for the random forest classifier are listed in Table 1

A random forest classifier classifies the input parameters by selecting the class with the highest probability. The probability value of the class “Good videos” is used as the overall quality score. The probability is in the scale between 0 and 1. The overall quality score is defined as 0 to 100 for uniformity with the previously calculated metrics scores and user-friendly interpretation. This scale conversion is achieved using Equation (15).

*o_s_* = 100 (*Probability* (*g_v_*)) (15)

where *o_s_* is overall quality score, and *g_v_* is “Good Videos”. Videos with overall quality scores greater than 50 are considered good videos. All other videos are considered as “manual editing required”.

### 5.8. Quality Feedback Generation

Providing quality feedback to users is a vital step in improving video quality for gait analysis. This methodology ensures that users receive actionable suggestions, enabling continuous improvement of video quality. Feedback is based on individual metric scores and helps users identify and address specific issues in their recordings. If any metric score is below 80, the algorithm provides targeted suggestions for improvement.

- Plane detection: If a sagittal or coronal view is not identified,
  ○ Suggestion for sagittal plane: “Ensure the camera is positioned above the pelvis height and parallel to the walkway (at a 90-degree angle to the side of the body).”
  ○ Suggestion for coronal plane: “Ensure the camera is at hip height and in front of the person.”
- Multiple persons score: If multiple persons are detected in the video, feedback recommends recording with only the person of interest in the frame.
  ○ Suggestion: “Ensure only the person of interest is in the frame during recording”.
- Multiple persons overlap score: If overlapping individuals are detected, then the video has more than one person in the frame. Feedback advises avoiding multiple persons in the frame.
  ○ Suggestion: “Ensure only the patient person of interest is in the frame during recording. Remove additional persons and avoid overlap if more than one person is required”.
- Zoom detection: If zoom artifacts are detected, feedback prompts users to maintain a stable camera.
  ○ Suggestion: “Keep the camera steady and do not use zoom while recording”.
- Videoclip length without zoom score: Video is too short.
  ○ Suggestion: “Ensure the person walks for at least 2 strides (4 steps). Ideally the person walks 5–6 strides, with the middle strides captured on video”.
- Resolution score: If the resolution score of the detected person is lower than 60 × 80 pixels, feedback suggests to adjust the camera distance to increase the number of pixels that define the person in the frame.
  ○ Suggestion: “Ensure the person occupies at least 50 percentage of total frame height. Adjust the camera distance accordingly”.

## 6. Validation Methods

Algorithm performance was assessed by comparing algorithm results with the manual classification.

The validation dataset for evaluating the multiple persons detection algorithm had 58 videos (videos from transverse plane excluded), including 38 videos with only one identifiable person for the entire video and 20 videos with two or more persons at the same time. Each video was manually reviewed for classification as single person or multiple persons.

The validation dataset for overlap detection had 58 videos, including 15 videos with overlap of one person over another person (overlap present set) and 43 videos without overlap of one person over another person (overlap absent set). Each video was manually reviewed and classified as overlap present or overlap absent.

The validation dataset for plane detection had 46 videos, in which 19 videos were coronal, 15 were sagittal, and 12 were transverse. Each video was manually reviewed to classify as coronal, sagittal, or transverse plane, and this manual classification was used as ground truth to validate algorithm classification performance.

The validation dataset for evaluating zoom detection had 26 videos, in which 14 videos had a zoom event (i.e., zooming-in or zooming-out), and 12 videos were without zoom events. Each video in the dataset was manually classified as having a zoom event or without a zoom event. Manual classification was used as ground truth to validate the identification of zoom events algorithm.

The validation dataset for evaluating the video segmentation algorithm had 30 videos. From the validation dataset, the algorithm extracts video segments without zoom. For videos from a coronal plane, there was one video segment for the person walking towards the camera and one video segment for the person walking away from the camera. For sagittal plane videos, there was one video segment for the person walking left to right and one video segment for person walking right to left. The extracted video segments were manually checked for the presence of zoom events, video segments of each walking orientation for every video, and a minimum video segment duration greater than 3 s. If there was a zoom event, then the video segment was flagged. This algorithm was evaluated using accuracy.

The overall video quality assessment algorithm was evaluated using a validation dataset that had 20 videos classified as “Good videos” and 24 videos as “Manual edit required”. Good videos can be used in an automated EVGS algorithm without any changes, and manual edit required videos cannot be used in automated EVGS algorithm without editing the video. Each video in the dataset was processed using the automated EVGS algorithm [3]. Based on the automated EVGS algorithm’s output—such as successful stride detection and the availability of all EVGS parameter scores—videos were subsequently manually annotated as either good videos or manual edit required videos. Random forest classifier performance was assessed by comparing the algorithm results with the manual classification.

## 7. Results

**Figure 5:**
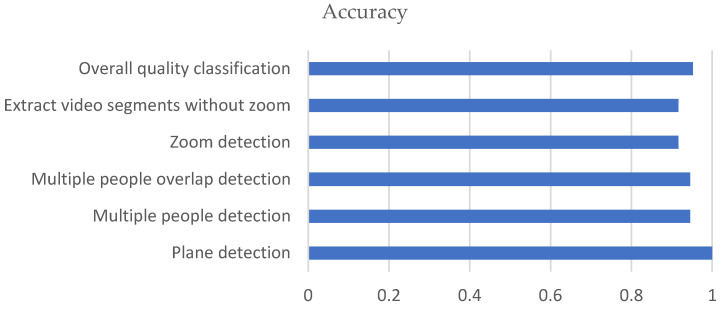
Outcome measure accuracy.

The accuracy results are shown in Figure 5.

### 7.1. Multiple People Detection

The validation dataset results had three misclassifications (Table 2 and Table 3), but all outcome measures were greater than 0.90.

### 7.2. Multiple People Overlap Detection

Algorithm performance for detecting overlapping persons is summarized in Table 4 and Table 5. The algorithm had strong performance, with a high recall (0.94) and specificity (0.95). Precision was the only score below 0.90. Only three videos were misclassified.

### 7.3. Plane Detection

As shown in Table 6, plane detection worked for all validation set classifications.

### 7.4. Zoom Detection

Zoom detection algorithm results are provided in Table 7 and Table 8. The algorithm had consistent results that ranged between 0.92 and 0.93. Only two videos were misclassified.

### 7.5. Extract Video Segments Without Zoom

Algorithm accuracy is summarized in Table 9. The algorithm processed 30 videos and extracted 60 video segments. Of these, only five clips were flagged, resulted in an accuracy of 92% in identifying video segments without zoom.

### 7.6. Overall Quality Classification

The Overall Video Quality Assessment framework was evaluated to assess its ability to classify gait videos as either good videos or manual edit required. The model demonstrated strong performance, underscoring its potential for practical applicability in automating video quality evaluation (Table 10 and Table 11).

## 8. Discussion

A video quality assessment method was developed to automatically score and identify appropriate feedback to the person capturing videos for use with tools such as EVGS to assess gait quality. The results from comparisons to manually scored videos demonstrated that this new approach could provide accurate classifications at a level that is acceptable for use in clinical environments.

The algorithm for detecting multiple people was successful for all but three videos, thereby demonstrating its effectiveness. The 0.97 specificity demonstrated that the algorithm minimized false-positive instances. These metrics indicated a system capable of maintaining good performance across diverse inputs. For the video with only one person classified as multiple people, the MoveNet pose estimation model output a high confidence value for two bounding box detections for the same person. False negatives occurred when a second person briefly moved into the image. The algorithm failed to find this presence because of low confidence scores, which sheds light on the challenge involved with identifying transient people in a dynamic video environment. It is noted that the person of interest was correctly identified in these frames (i.e., no effect on outcomes).

For multiple persons overlap detection, the algorithm achieved 94% sensitivity and 95% specificity, indicating its effectiveness in detecting overlapping. Moreover, the Matthews Correlation Coefficient (MCC) of 0.87 emphasizes its ability to deal with imbalanced datasets, where overlapping situations are much less common than non-overlapping frames. A detailed analysis of the mistakes showed two cases where the algorithm struggled. In the first case, a false positive was made when the MoveNet pose estimation model output a high confidence value for two bounding box detections for the same person. A false negative was made during an actual overlap scenario, where the confidence scores assigned to the second person were low and the overlap with the person of interest was very short. As pose detection models mature, multiple persons detection errors should reduce.

The algorithm correctly classified all videos as sagittal, coronal, or transverse. The inclusion of challenging edge cases, such as videos containing zoom events and multiple people, confirmed the algorithm’s robustness.

The algorithm caught almost all zoom events. Moreover, the algorithm had few false positives, ensuring that non-zoom events were not misclassified. The F1-score of 0.93 emphasized the balance between precision and recall, further ascertaining the effectiveness of the algorithm in the reduction of false positives and false negatives. Of the errors, false positives were found in videos labelled as non-zoom, where small variations in bounding box size led to misclassification. These were attributed to factors such as limping that caused changes in the person’s estimated height and small variations in the keypoints. Similarly, false negatives were recorded in videos where there were zoom events. In both cases, the zooming action was gradual and subtle, causing changes in the bounding box size that fell below the algorithm’s detection threshold. These false negatives highlight the difficulty of the algorithm in recognizing small zoom events; however, these small zoom events should have a minimal effect on automated EVGS scoring.

The algorithm effectively identified video segments longer than 3 s and without zoom events. The errors that occurred could be attributed to cases where the person walked towards the camera when zooming-in, and for the person walking away from camera and zooming-out, especially when the zoom event was subtle, for example, when the change in a person’s height due to zooming was smaller than the change in height caused by the person walking toward or away from the camera. Other than this subtle zoom, the algorithm can detect sharp zoom events effectively (sudden change in person’s height).

The random forest classifier achieved high performance when providing an overall gait video quality classification. The system’s success can be attributed to the use of multiple quality metrics that enabled a thorough evaluation of the video characteristics. Instead of only classified labels like the video is good or manual edit required, probability-based results will help clinicians to understand video quality on a scale so that they can interpret if the video is very bad or closer to acceptable. Further research is needed to verify if clinicians would use this probability score or just the binary classification. Both the overall classification and score (0–100) would be provided to the user. Two videos were misclassified (videos that needed hand editing were wrongly determined to be acceptable). This occurred when videos barely passed thresholds for individual criteria. Also, false negatives emerged where slight deviations in one or more metrics resulted in the misclassification of good videos as needing manual edits. These borderline cases suggest areas where improvement is possible.

The automated video quality assessment method in this research could dramatically reduce manual labor in pre-processing gait videos because the integration of different metrics into one framework makes model-based evaluations uniform and objective. Other opportunities to improve the algorithm could include adding complementary features, such as keeping track of motion stability and lighting consistency. Refining thresholds for individual metrics, based on deeper analysis of misclassified videos, could further reduce these small error rates.

## 9. Conclusions

This research presented an advancement in video quality assessment related to automated gait analysis. By addressing key video quality issues such as plane misalignment, the presence of multiple individuals, subject occlusion, zoom artifacts, and insufficient resolution, the proposed system ensures that only clinically viable videos are forwarded for automated EVGS scoring. Leveraging the MoveNet Multipose Lightning pose estimation model, the framework accurately detects and tracks the person of interest across frames, providing reliable input for subsequent gait evaluation tasks.

Integrating individual quality metrics, the final random-forest-based quality assessment classifier achieved high accuracy in determining whether a video was suitable for automated EVGS analysis or required manual intervention. In addition to binary quality classification, the framework also provides interpretable and actionable feedback, guiding users in rectifying video quality issues. This feature contributes to a more efficient and user-friendly gait analysis workflow by reducing the reliance on manual screening and improving video acquisition practices in clinical environments.

Overall, this research represents an important step forward in the automation of video-based gait analysis by ensuring the reliability of input data. Future research will focus on broadening the framework’s capabilities to detect a wider range of video artifacts and integrating the system into a fully automated EVGS scoring pipeline, thus enhancing the scalability and clinical utility of gait assessment technologies.

## Figures and Tables

**Table 1:** Fine-tuned hyperparameters using grid search approach.

Hyperparameters	Fine-Tuned Value
Number of estimators	50
Maximum depth	None
Minimum samples required to split a node	2
Minimum samples required at leaf node	1
Bootstrap	False

**Table 2:** Confusion matrix of multiple persons detection.

	True Multiple People Detected	True Multiple People Not Detected
Predicted Multiple Persons Detected	18	1
Predicted Multiple Persons Not Detected	2	37

**Table 3:** Performance metrics for multiple persons detection.

Accuracy	Precision	Sensitivity	F1 Score	Specificity	MCC
0.95	0.95	0.90	0.92	0.97	0.88

**Table 4:** Confusion matrix of multiple persons overlap detection.

	True Multiple Persons Overlap Detected	True Multiple Persons Overlap Not Detected
Predicted Multiple Persons Overlap Detected	14	2
Predicted Multiple Persons Overlap Not Detected	1	41

**Table 5:** Performance metrics for multiple persons overlap detection.

Accuracy	Precision	Sensitivity	F1 Score	Specificity	MCC
0.95	0.88	0.94	0.91	0.95	0.87

**Table 6:** Performance metrics for plane detection.

Accuracy	Precision	Sensitivity	F1 Score	Specificity	MCC
1.0	1.0	1.0	1.0	1.0	1.0

**Table 7:** Confusion matrix of zoom detection.

	True Zoom Detected	True Zoom Not Detected
Predicted Zoom Detected	13	1
Predicted Zoom Not Detected	1	11

**Table 8:** Performance metrics for zoom detection.

Accuracy	Precision	Sensitivity	F1 Score	Specificity	MCC
0.92	0.93	0.93	0.93	0.92	0.85

**Table 9:** Accuracy of the algorithm.

No. of Videos	No. of Video Segments Extracted	No. of Flagged Video Segments	Accuracy
30	60	5	0.92

**Table 10:** Confusion matrix of overall video quality classification.

	True “Good Videos”	True “Manual Edit Required Videos”
Predicted “Good videos”	23	1
Predicted “Manual edit required videos”	1	19

**Table 11:** Performance metrics for overall video quality classification.

Accuracy	Precision	Sensitivity	F1 Score	Specificity	MCC
0.95	0.96	0.96	0.96	0.95	0.91

## Data Availability

The dataset in this research includes sensitive human participant data containing identifiable and personal medical information. The dataset cannot be shared publicly.

## References

[1] Whittle M.W. (2007). Gait Analysis: An Introduction.

[2] Middleton A., Fritz S.L. (2013). Assessment of Gait, Balance, and Mobility in Older Adults: Considerations for Clinicians. Curr. Transl. Geriatr. Exp. Gerontol. Rep..

[3] Ramesh S.H., Lemaire E.D., Tu A., Cheung K., Baddour N. (2023). Automated Implementation of the Edinburgh Visual Gait Score (EVGS) Using OpenPose and Handheld Smartphone Video. Sensors.

[4] Ramesh S.H., Lemaire E.D., Cheung K., Tu A., Baddour N. Automated Stride Detection from OpenPose Keypoints Using Handheld Smartphone Video. Proceedings of the 2023 IEEE Sensors Applications Symposium (SAS).

[5] Rodrigues R., Lévêque L., Gutiérrez J., Jebbari H., Outtas M., Zhang L., Chetouani A., Al-Juboori S., Martini M.G., Pinheiro A.M. (2024). Objective quality assessment of medical images and videos: Review and challenges. Multimed. Tools Appl..

[6] Razaak M., Martini M.G. Medical Image and Video Quality Assessment in E-Health Applications and Services. Proceedings of the 2013 IEEE 15th International Conference on e-Health Networking, Applications and Services (Healthcom 2013).

[7] Leszczuk M. (2011). Assessing Task-Based Video Quality—A Journey from Subjective Psycho-Physical Experiments to Objective Quality Models. Multimedia Communications, Services and Security, Proceedings of the 4th International Conference, MCSS 2011, Krakow, Poland, 2–3 June 2011.

[8] Raj A., Tiwari A.K., Martini M.G. (2019). Fundus image quality assessment: Survey, challenges, and future scope. IET Image Process..

[9] Li Y., Po L.-M., Feng L., Yuan F. No-Reference Image Quality Assessment with Deep Convolutional Neural Networks. Proceedings of the 2016 IEEE International Conference on Digital Signal Processing (DSP).

[10] Liang J., Cao J., Sun G., Zhang K., Van Gool L., Timofte R. SwinIR: Image Restoration Using Swin Transformer. Proceedings of the 2021 IEEE/CVF International Conference on Computer Vision Workshops (ICCVW).

[11] Wiegand T. (2003). Draft itu-t Recommendation and Final Draft International Standard on Joint Video Specification. H. 264/ISO/IEC 14496-10 AVC, JVT-G050. https://cir.nii.ac.jp/crid/1572824500866259584.

[12] Wu H., Chen C., Liao L., Hou J., Sun W., Yan Q., Lin W. (2023). DisCoVQA: Temporal Distortion-Content Transformers for Video Quality Assessment. IEEE Trans. Circuits Syst. Video Technol..

[13] Wu H., Zhang E., Liao L., Chen C., Hou J., Wang A., Sun W., Yan Q., Lin W. (2023). Exploring Video Quality Assessment on User Generated Contents from Aesthetic and Technical Perspectives. Proceedings of the 2023 IEEE/CVF International Conference on Computer Vision (ICCV).

[14] Mirkovic M., Vrgovic P., Culibrk D., Stefanovic D., Anderla A. (2014). Evaluating the Role of Content in Subjective Video Quality Assessment. Sci. World J..

[15] Zhang L., Cavaro-Ménard C., Callet P.L. Key Issues and Specificities for the Objective Medical Image Quality Assessment. Proceedings of the Sixth International Workshop on Video Processing and Quality Metrics for Consumer Electronics VPQM 2012.

[16] Welikala R.A., Fraz M.M., Foster P.J., Whincup P.H., Rudnicka A.R., Owen C.G., Strachan D.P., Barman S.A., UK Biobank Eye and Vision Consortium (2016). Automated retinal image quality assessment on the UK Biobank dataset for epidemiological studies. Comput. Biol. Med..

[17] Rodrigues R., Pinheiro A.M.G. (2019). Segmentation of Skeletal Muscle in Thigh Dixon MRI Based on Texture Analysis. arXiv.

[18] Alais R., Dokládal P., Erginay A., Figliuzzi B., Decencière E. (2020). Fast macula detection and application to retinal image quality assessment. Biomed. Signal Process. Control..

[19] Del Pilar Duque Orozco M., Abousamra O., Church C., Lennon N., Henley J., Rogers K.J., Sees J.P., Connor J., Miller F. (2016). Reliability and validity of Edinburgh visual gait score as an evaluation tool for children with cerebral palsy. Gait Posture.

[20] Movenet Multipose Lightning. https://www.kaggle.com/models/google/movenet/tensorFlow2/multipose-lightning.

[21] Ye R.Z., Subramanian A., Diedrich D., Lindroth H., Pickering B., Herasevich V. (2022). Effects of Image Quality on the Accuracy Human Pose Estimation and Detection of Eye Lid Opening/Closing Using Openpose and DLib. J. Imaging.

